# Nefopam downregulates autophagy and c-Jun N-terminal kinase activity in the regulation of neuropathic pain development following spinal nerve ligation

**DOI:** 10.1186/s12871-018-0559-8

**Published:** 2018-07-27

**Authors:** Seon-Hee Oh, Myung Ha Yoon, Kyung Joon Lim, Byung Sik Yu, In Gook Jee, Ki Tae Jung

**Affiliations:** 1 School of Medicine, Chosun University, 309 Pilmundaero, Dong-gu, Gwangju, 501-759 South Korea; 2 Department of Anesthesiology and Pain Medicine, Medical School, Chonnam National University, 42 Jebongro, Donggu, Gwangju, 501-757 South Korea; 3 Department of Anesthesiology and Pain Medicine, School of Medicine, Chosun University, Chosun University Hospital, 365 Pilmun-daero, Dong-gu, Gwangju, 61453 South Korea; 4 Department of Anesthesiology and Pain Medicine, Chosun University Hospital, 365 Pilmun-daero, Dong-gu, Gwangju, 61453 South Korea

**Keywords:** Autophagy, JNK, Nefopam, Neuropathic pain, Rat

## Abstract

**Background:**

Neurodegeneration is associated with changes in basal cellular function due to the dysregulation of autophagy. A recent study introduced the involvement of autophagy during spinal nerve ligation (SNL). Nefopam has shown potential for reducing neuropathic pain, but the underlying mechanisms are unknown. Here, we investigated the effects of nefopam on neuropathic pain development following SNL, focusing on the involvement of autophagy.

**Methods:**

The functional role of nefopam in capsaicin-induced autophagy was assessed by human glioblastoma M059 K cells. The neuropathic pain model was used to determine whether the effect of nefopam on pain control was mediated through autophagy control. Neuropathic pain was induced by L5 and L6 SNL in male rats randomized into three groups: Group S (sham-operated), Group C (received normal saline), and Group E (received nefopam). A behavioral test using a von Frey was examined. Expression changes of autophagy in response to nefopam was analyzed in spinal cord tissues (L4-L6) by immunoblotting and immunohistochemistry.

**Results:**

The paw withdrawal threshold examined on days 3, 5, 7, and 14 post-SNL was significantly higher in Group E than in Group C. SNL increased the levels of microtubule-associated protein 1 light chain 3B (LC3B-1), with concomitant reduction of sequestosome 1 (SQTSM1/p62), compared with Group S, indicating that SNL induced autophagy. These effects were reversed by nefopam injection, and the results were confirmed by immunohistochemistry for LC3-I/II. Furthermore, SNL-mediated JNK activation was markedly decreased following nefopam injection. Hematoxylin and eosin staining on Day 14 post-SNL revealed that SNL caused lymphocyte infiltration and oligodendrocyte localization in the substantia gelatinosa of the dorsal gray horn, which were reduced by nefopam injection.

**Conclusion:**

Collectively, the mode of action of nefopam on neuropathic pain appears to be associated with downregulation of phospho-JNK and autophagy, as well as modulation of the immune response.

## Background

Neuropathic pain was previously thought to be the consequence of changes in the activity of neuronal systems, immune cells, and immune cell-derived inflammatory cytokines [1]. However, a recent study reported that an imbalance in the autophagic process following nerve injury can lead to changes in basal cell functions closely associated with neurodegeneration [2].

According to Berliocchi et al. [2], completion of basal autophagy and autophagosome turnover are blocked as a consequence of spinal nerve ligation (SNL), and this may be associated with neurodegeneration and the development of neuropathic pain. When programmed cell death is functionally disordered, various apoptotic stimuli activate autophagy and c-Jun N-terminal kinase (JNK), resulting in the induction of autophagic cell death, which can lead to neurodegeneration [3, 4]. JNK activation is closely related to the development of neuropathic pain. Following nerve injury, JNK is rapidly activated primarily in small diameter C-fiber neurons [5, 6]. Furthermore, JNK inhibitors prevent the development of mechanical allodynia after SNL [5, 7]. A recent study on the effects of curcumin also demonstrated the prevention of chronic neuropathic pain by suppression of JNK phosphorylation [8].

Nefopam is a centrally acting analgesic that has similar action to triple neurotransmitter reuptake inhibitors and anticonvulsants. Recently, nefopam has been suggested for the treatment of neuropathic pain because it has analgesic properties [9–13]. Prophylactic administration of nefopam had a preventive effect on the development of neuropathic pain after chronic constriction injury of the sciatic nerve [14]. However, the molecular mechanisms through which nefopam exerts these anti-neuropathic pain effects are not completely understood. Therefore, we investigated the preventive effects of nefopam on the development of neuropathic pain following SNL, focusing on the involvement of autophagy and JNK activation.

## Methods

### Cell culture and chemicals for in vitro experiments

Human glioblastoma M059 K cells (ATCC^®^ CRL-2365™) were maintained in DMEM (Gibco BRL, Grand Island, NY) supplemented with heat-inactivated 10% fetal bovine serum, 50 μg/ml penicillin, and 50 μg/ml streptomycin at 37 °C in a 5% CO_2_–95% air humidified incubator. Capsaicin, SP600125, and thapsigargin were obtained from Sigma (St. Louis, MO) and Calbiochem (La Jolla, CA USA), respectively. Other chemicals used were of the purest grade available from Sigma (St. Louis, MO).

### Animal preparation

This study was approved by the Institutional Animal Care and Use Committee of Chonnam National University (CNU IACUC-H-2015-26) and followed the International Association for the Study of Pain guidelines on ethical standards for the investigation of experimental pain in animals [15]. Experiments were performed on male Sprague–Dawley rats (specific pathogen free) weighing 100–120 g. Rats were purchased from Damul Science (Daejeon, Korea). The animals were raised in cages in a temperature-controlled room (20 to 23 °C) with a 12-h light/dark cycle and free access to food and water.

### Groups and induction of neuropathic pain

Rats were randomized into three groups (total *n* = 36, *n* = 12 in each group) according to the random numbers generated by a computer. Rats in the sham group (group S) underwent a sham operation without SNL. While, rats in the control and experimental groups (groups C and E) received SNL for the induction of neuropathic pain Groups and induction of neuropathic pain [16, 17]. Experiments were carried out after confirming that the rats had no neurological abnormality. After sevoflurane anesthesia, L5–S2 spine were dissected and the left L5 and L6 spinal nerves were tightly ligated.

### Drug administration

Those in group S had just sham operation. Rats in the group C were administered normal saline for 7 days following the SNL procedure. Those in group E were administered 30 mg/kg of nefopam hydrochloride (Acupan^®^, Pharmbio, Seoul, Korea) intraperitoneally for 3 days immediately following the SNL procedure [13, 14].

### Spinal cord sampling

On the 14th day, rats were euthanized by decapitation under sevflurane overdose anesthesia. Then, the spinal cord was isolated by flushing with ice-cold phosphate-buffered saline from the caudal end of the vertebral column. After obtaining the ipsilateral dorsal spinal cord at L4–L6 by a cut in the spinal cord at the midline, the tissue was immediately stored at − 70 °C by liquid nitrogen until homogenization.

### Western blotting

Tissue samples were homogenized in a lysis buffer with a Dounce homogenizer, further lysed with RIPA buffer containing protease inhibitor cocktail (Sigma). Cell lysates was quantified for protein content, and separated by SDS-PAGE in 12–15% acrylamide gels, transferred to polyvinylidene difluoride membranes (Millipore, Billerica, MA, USA), and then immunoblotted with corresponding antibodies. Phospho-JNK (#9251), JNK (#9258), mammalian target of rapamycin (mTor, #2972), and phospho-mTor (#2971) were obtained from Cell Signaling (Danvers, MA, USA). Anti-rabbit polyclonal atg8/LC3 antibody (#3868) was obtained from Cell Signaling (Irvine, CA, USA). TNF-α (sc-52,746) and β-actin (sc-70,319) were purchased from Santa Cruz Biotechnology (Santa Cruz, CA, USA). The bands were visualized using chemiluminescence Western Blotting Detection Reagents (Millipore), and quantified with ImageJ densitometry software (National Institutes of Health, Bethesda, MD).

### Immunohistochemistry

After conventional dewaxing and microwave antigen retrieval in 10 mM sodium citrate buffer (pH 6) for 10 min, sections were incubated with microtubule-associated protein 1 light chain (LC3) antibody (1:50) (#3868) overnight at 4 °C. A negative control without primary antibody was performed for each specimen. Endogenous peroxidase activity was prevented by incubating the sections for 15 min in 0.3% H_2_O_2_. Immunohistochemistry procedures were performed using the Polink-2 AP broad detection kit according to the supplier’s protocol (Life Science Division, WA, USA). The slides were counterstained with hematoxylin and mounted with an immunohistomounting medium (Abcam, Cambridge, MA, USA). Histological changes in spinal cord tissues were observed under a microscope (Nikon, TE300, Japan).

### Hematoxylin and eosin staining of paraffin-embedded tissues

Spinal cord tissues were cut into approximately 0.5 cm length pieces vertically in the center of L5 and were fixed in neutral buffered formalin, dehydrated, embedded in paraffin, and sectioned. The sections were deparaffinized in xylene, rehydrated in an ethanol gradient, and stained with hematoxylin and eosin (H & E). Pathological changes in spinal cord tissues were observed under an optical microscope (Nikon, TE300, Japan).

### Assessment of mechanical allodynia

Mechanical allodynia was assessed by paw withdrawal threshold (PWT) measured with von Frey filaments (Stoelting, Wood Dale, IL, USA). After acclimation in the laboratory environment for 30 min, mechanical stimulation was applied to the plantar surface of the hind paw vertically for 5 s with a series of eight von Frey filaments (0.4, 0.7, 1.2, 2.0, 3.6, 5.5, 8.5, and 15 g). Abrupt withdrawal or flinching of the hind paw were regarded as a positive response and PWT was calculated by the up and down method [18]. The cut-off value was a negative response to 15 g. A series of tests were conducted on the 3rd, 5th, 7th, and 14th day following SNL.

### Statistical analysis

Data were expressed as the mean ± standard error of the mean. The results of the behavioral experiments were analyzed by a repeated measures one-way analysis of variance and Scheffe’s post-hoc test. The differences between groups were analyzed by t-test. Non-parametric data were analyzed by the Kruskal-Wallis test followed by Scheffe’s post-hoc test. Results with *p*-values < 0.05 were considered statistically significant. A two tailed t test was performed for comparisons of densitometry between the groups.

## Results

### Nefopam suppressed JNK-mediated autophagy induced by capsaicin

**Fig. 1:**
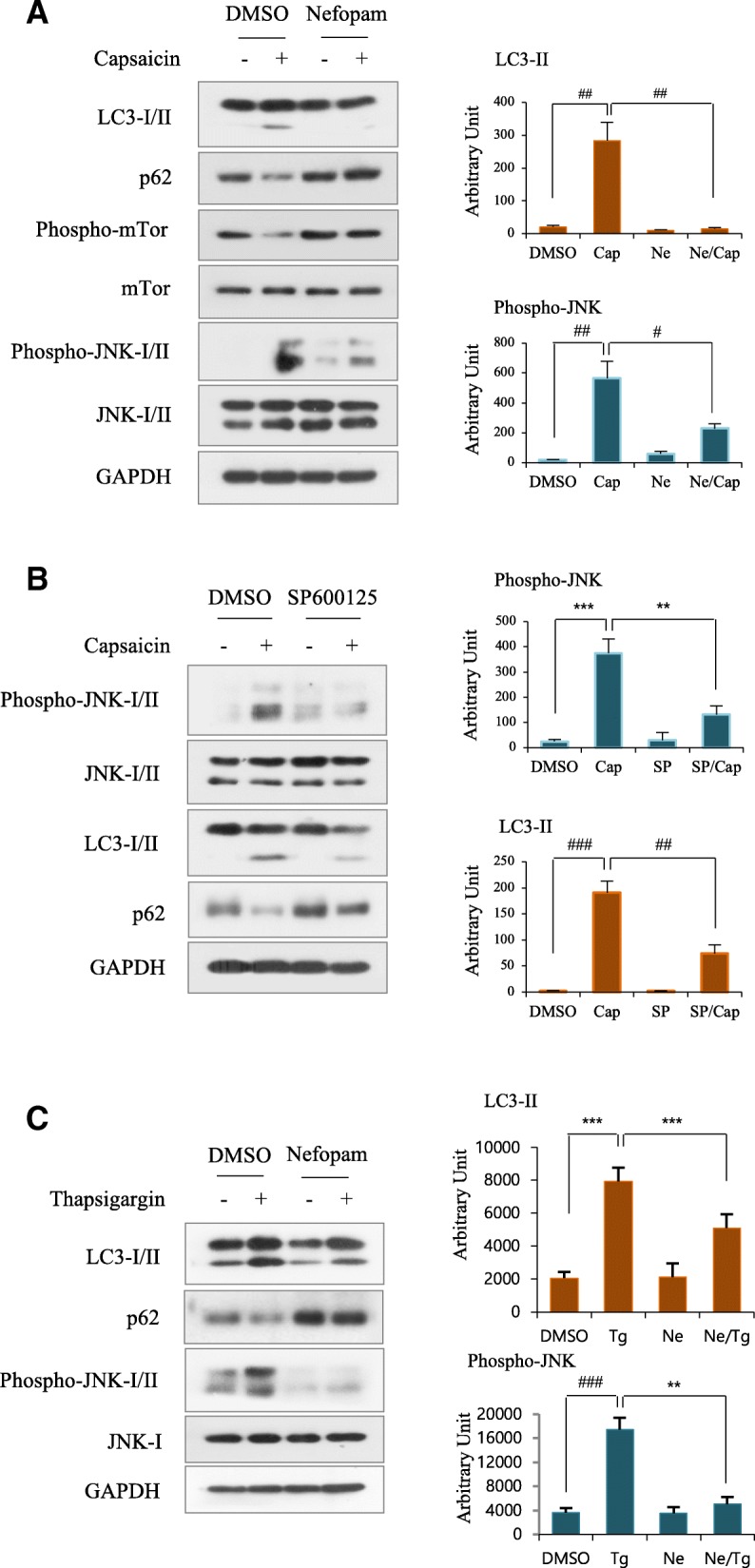
Nefopam downregulates capsaicin-induced LC3-II and phospho-JNK in M059 K cells. **a** Cells were pretreated with nefopam (50 μM) and continuously exposed capsaicin (250 *μ*M) for 18 h. Cell lysates were immunoblotted for indicated proteins. **b** M059 K cells were pretreated with SP600125 (10 *μ*M) and continuously exposed capsaicin (250 *μ*M) for 18 h. GAPDH was used as the loading control. **c** Cells were pretreated with nefopam (50 *μ*M) and continuously exposed thapsigargin (Tg, 4 μg/ml) for 18 h. Cell lysates were immunoblotted for indicated proteins. All immunoblot data are representative of at least three independent experiments. Bar graphs show densitometrical analysis for LC3-II and phospho-JNK-II. A two tailed t test was performed for comparisons between groups (*n* = 3). ^#^*p* < 0.01, ^##^*p* < 0.002, ^###^*p* < 0.0002 **p < 0.005, ***p < 0.0005

Capsaicin, the pungent ingredient in hot chili peppers, is a selective agonist of transient receptor potential cation channel subfamily V member 1 (TRPV1), which is involved in pain sensation [19]. Capsaicin exposure induced autophagy in M059 K cells, which was confirmed by reduction of p62 (SQTSMI/sequestosome 1), an autophagy adaptor molecule, which was upregulated by nefopam pretreatment. Furthermore, capsaicin exposure induced downregulation of mTor, and that was upregulated by nefopam pretreatment, indicating that nefopam might suppress capsaicin-induced autophagy via mTor activation. Additionally, capsaicin activated the mitogen-activated protein kinase (MAPK) JNK, which was suppressed by pretreatment with nefopam (Fig. 1a). Next, we examined whether JNK activation is involved in autophagy induction in capsaicin-exposed cells. For this purpose, we used a pharmacological JNK inhibitor, SP600125. As expected, pretreatment with SP600125 before adding capsaicin suppressed capsaicin-induced LC3-II, an autophagy marker (Fig. 1b). To further substantiate the inhibitory role of nefopam in JNK-mediated autophagy we performed additional experiment by using a selective endoplasmic reticulum stress inducer, thapsigargin (Tg) in normal kidney cells (MES13E: SV40 MES 13, ATCC® CRL-1927™). Treatment with Tg induced JNK activation and autophagy, which were suppressed by pretreatment with nefopam (Fig. 1c). Collectively, these results indicat that nefopam may suppress autophagy by regulating signaling pathway implicated with mTor and MAPK JNK.

### Nefopam reduced mechanical allodynia and lymphocyte infiltration following SNL

**Fig. 2:**
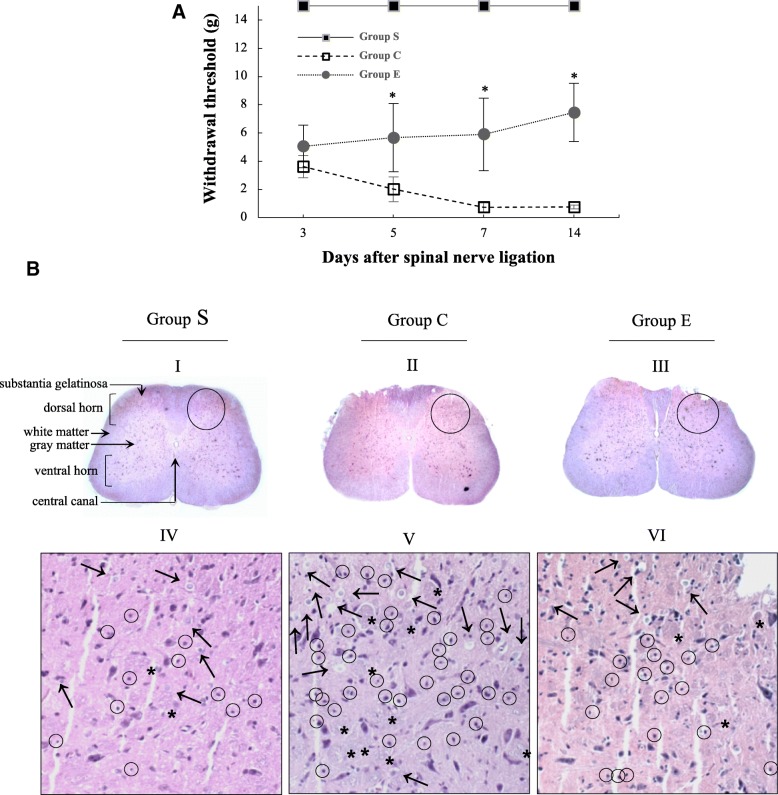
Mechanical allodynia and histological changes following SNL. **a** Paw withdrawal threshold over time. The paw withdrawal thresholds were observed on the 3rd, 5th, 7th, and 14th day post spinal nerve ligation (SNL). The development of neuropathic pain was confirmed with decreased withdrawal thresholds of the rats from group C and E compared to the group S throughout the observation periods (*p* < 0.001). The withdrawal thresholds are significantly higher in rats from group E than in those from group C from the 5th day post SNL (*p* < 0.001). *p < 0.05 compared with group C. **b** Hematoxylin and eosin staining in the the spinal cord sections. Sections from group S (I, IV) show only a small number of lymphocytes (small circles), normal oligodendrocytes (arrows) with prominent perinuclear halos, and vacuoles containing poorly stained material (stars). Sections from group C (II, V) show lymphocytes infiltration and many oligodendrocytes localized in the substantia gelatinosa of the posterior gray horn. Sections from group E (III, VI) show a reduced number of lymphocytes and oligodendrocytes than those in group C. Group S, Sham-operated; Group C, saline-treated after SNL; Group E, nefopam-treated after SNL. Original magnification, X200. →, Oligodendrocytes; o, Lymphocytes; *, Vacuoles

Mechanical allodynia was confirmed in all rats from Group C (received normal saline) and Group E (received nefopam) 3 days after the SNL procedure. Rats from Group S (sham-operated) showed no mechanical allodynia (*n* = 12). The PWT was significantly reduced in rats from Groups C and E compared with those in Group S. The PWT of rats from Group E was higher than that of rats from Group C during the observation period (*n* = 12 for each group). However, significant differences were recorded on Day 5 post-SNL (Fig. 2a). These results indicate that nefopam decreased pain sensation induced by SNL.

To observe histopathological changes on Day 14 after surgery, spinal cord tissue sections were subjected to hematoxylin and eosin staining, and histological changes were observed in the dorsal gray matter region (Fig. 2b, I, II, and III, large circles). In sections from Group S, only a small number of lymphocytes were observed (Fig. 2b, IV, small circles). Normal oligodendrocytes (arrows) with prominent perinuclear halos were also identified. Furthermore, vacuoles containing poorly stained material were observed (Fig. 2, IV, V, and VI, stars). In contrast, tissues from Group C showed lymphocyte infiltration and many oligodendrocytes localized mainly in the substantia gelatinosa of the posterior gray horn (Fig. 2b, V). Tissues from Group E had reduced numbers of lymphocytes and oligodendrocytes compared to those in the SNL group; however, this was still higher than the numbers of these cells in Group S (Fig. 2, VI). Collectively, these data indicate that nefopam suppressed pain-mediated immune responses.

### Immunoreactivity of LC3-I/II in spinal cord tissue sections

**Fig. 3:**
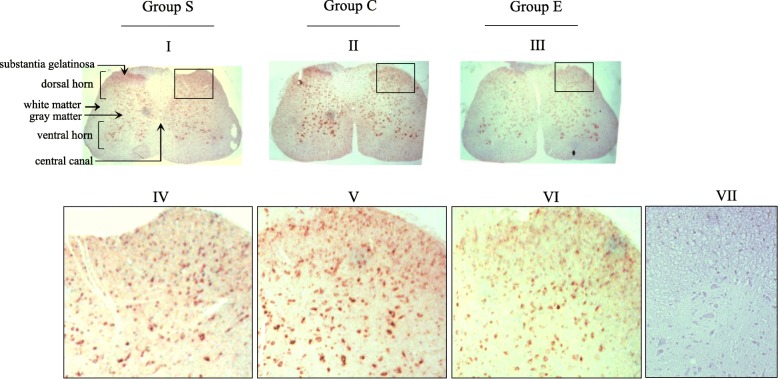
Immunohistochemical study for LC3-I/II expression in the spinal cord sections. In group S (I, IV), the immunoreactivity of LC3-I/II is visible in most cells (polygonal or ovoid) localized in the dorsal gray matter of ipsilateral spinal cord sections, which is further enhanced in group C (II, V), but suppressed in group E (III, VI). Antibody diluents, used as a negative control for LC3-I/II (VII). Group S, sham-operated; Group C, saline-treated after SNL; Group E, nefopam-treated after SNL. Original magnification, X 200

To examine whether autophagy is involved in the actions of nefopam in pain mitigation, we analyzed the immunoreactivity of LC3-I/II via immunostaining. Cells positive for the LC3-I/II antibody were localized mainly in gray matter across all sections. Strong positive signals were localized in the cell bodies of the neurons residing in the ventral horn. Furthermore, immunoreactivity revealed differences between the dorsal gray horn and the ventral gray horn. We thus compared LC3-I/II immunoreactivity in the dorsal gray matter of the spinal cord ipsilateral with the ligation side. In Group S, the immunoreactivity of LC3-I/II was observed in most cells (polygonal or ovoid) localized in the dorsal gray matter (Fig. 3, I and IV), which was further enhanced in the tissues from Group C (Fig. 3, II and V), but was suppressed by nefopam injection (Fig. 3, III and VI). Antibody diluents, used as a negative control for LC3-I/II, did not exhibit LC3-I/II immunoreactivity (Fig. 3, VII). These results suggest the involvement of LC3-I/II in nefopam action on neuropathic pain.

### LC3-I/II expression and JNK activation in nefopam-mediated pain

**Fig. 4:**
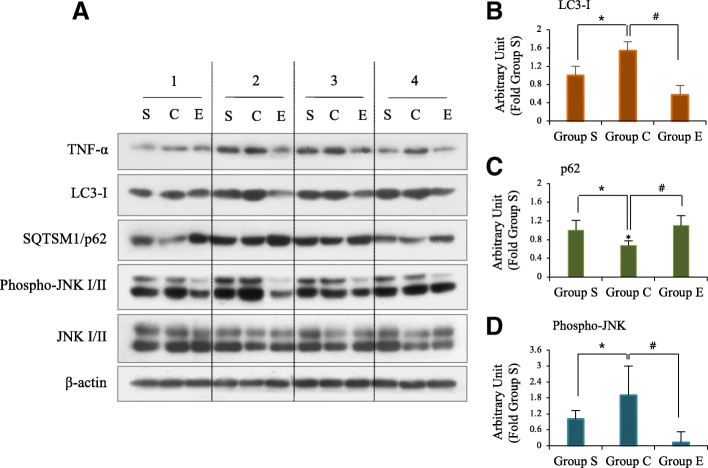
The effects of nepofam on SNL-mediated autophagy and JNK activation. **a** Spinal cord tissue homogenates obtained from on 14 day after SNL, and analyzed for indicated proteins by Western blot analysis. β-actin was used as the loading control. Group S, sham-operated; Group C, saline-treated after SNL; Group E, nefopam-treated after SNL. **b**-**d** Densitometrical analysis for LC3-I, p62, and phospho-JNK-II. A two tailed t test was performed for comparisons between groups (*n* = 4). * *p* < 0.05, ^#^*p* < 0.01

Our in vitro experiments revealed that the expression of phospho-JNK and LC3-II induced by capsaicin, a pain sensation inducer, were suppressed by nefopam (Fig. 1). We thus investigated the action of nefopam on JNK and autophagy activation with an in vivo experiment. Tumor necrosis factor alpha (TNF-α) is known to play a pivotal role in neuropathic pain [20]. Consistently, SNL resulted in TNF-α production, which was suppressed by nefopam injection. The immunohistochemical staining for LC3-I/II expression was further confirmed by western blot analysis (Fig. 4a), and LC3-I expression was evaluated by densitometry (Fig. 4b). We did not detect membrane-bound LC3-II with the antibodies used. However, on Day 14 post-SNL, we observed an increase in LC3-I with a concomitant decrease in the autophagy adaptor molecule, p62, indicating that autophagy was increased by SNL. Furthermore, SNL-mediated LC3-I levels decreased, and p62 levels increased following nefopam injection (Fig. 4a and c), indicating that SNL-mediated autophagy was inhibited by nefopam.

We then examined MAPK JNK, which is known to be related to neuropathic pain following SNL [7]. SNL resulted in JNK activation compared with rats from Group S, as demonstrated by western blotting with phospho-antibody on Day 14 post-SNL, and JNK was markedly suppressed by nefopam, as evaluated by densitometry (Fig. 4a, d). However, the levels of phospho-JNK in one of the rats demonstrated less change. The level of phospho-JNK in Group S was high, indicating that JNK may play a role at the non-stressed basal level.

## Discussion

In the present study, we demonstrated that nefopam injection following SNL downregulated JNK activation and autophagy and increased PWT. To the best of our knowledge, this is the first study to demonstrate the effects of nefopam on the regulation of MAPK JNK activation and autophagy.

Recently, there was a suggestion about the possibility of nefopam in the treatment of neuropathic pain [9], because the analgesic mechanisms of nefopam are related to not only the inhibition of monoamine reuptake but also the inhibition of NMDA receptors [21]. The excitatory neurotransmitter, glutamate, acts via N-methyl-D-aspartate receptors (NMDARs). NMDARs might control the neurochemical axis involved in neuronal function through MAPK activation [22]. When NMDA receptors are stimulated, MAPK is activated by tyrosine phosphorylation [22]. In stressful conditions such as nerve injury, JNK activating Jun transcription factor and the p38 MAPK are stimulated [22]. Previous reports showed that neuropathic pain following SNL may be associated with autophagic activity [2, 23], and neurodegeneration in a results of JNK-mediated autophagic cell death [3, 4]. Therefore, we attempted to determine the role of nefopam in the prevention of neuropathic pain development in relation to autophagy and JNK activation.

For this purpose, we conducted the study using M059 K cells which can demonstrate the capsaicin induces autophagy which is involved in pain sensation, firstly [24]. Pain could act as a stress to cells, and a variety of stresses induce autophagy and stress-activated MAPK JNK activation [24]. We used capsaicin, an agonist of the non-selective cation channel TRPV1, which depolarizes cells and leads to a painful sensation when activated [25]. Furthermore, capsaicin is known to induce autophagy via JNK activation [24]. In the present study, we found that capsaicin induced autophagy and mTor inhibition in neuronal blastoma M059 K cells, indicating that capsaicin-induced autophagy might be dependent on mTor pathway, which was inhibited by nefopam. Moreover, capsaicin activated JNK, which was associated with autophagy induction, as determined by a pharmacological inhibitor of JNK. The causative relationship between autophagy and nefopam was tested by using autophagy inducer, Tg; nefopam suppressed Tg-mediated autophagy and JNK activation. Therefore, nefopam may suppress autophagic activation by inhibiting the mTor signaling pathway and JNK. These in vitro data suggested the possibility that pain mitigation by nefopam may depend on JNK-mediated autophagy. Therefore, our present results suggest a possibility that nefopam may act on pain relief via suppression of JNK-mediated autophagy.

Then, the effect of nefopam on the development of neuropathic pain was confirmed in vivo study. Administration of intraperitoneal nefopam (30 mg/kg, 3 days post SNL) increased the withdrawal threshold and downregulated activation of JNK and autophagy. Immunohistochemistry for LC3-I/II, an autophagy marker, revealed that SNL-induced LC3-I/II immunoreactivity decreased following nefopam injection. Strong positive staining was localized in the cell body of the neurons, indicating that the action of nefopam might occur via affecting on function of neuron. Furthermore, a change in the immunoreactivity of LC3-I/II was revealed in the dorsal horn of the gray matter, indicating that autophagy may affect sensory neurons rather than motor neurons. However, our hypothesis requires further clarification. Western blot analysis of spinal cord tissue lysates revealed that SNL activated JNK and autophagy, which were mitigated by nefopam. Therefore, our in vitro results are consistent with previous reports showing that SNL-mediated JNK activation was inhibited by nefopam [5, 6].

Glial cells including oligodendrocytes, astrocytes, and microglia, are known to play important roles in the pathology of neuroinflammation and neuropathic pain [26]. In the present study, histological analysis revealed that the number of oligodendrocytes in the substantia gelatinosa of the dorsal gray horn of rat underwent SNL increased than in sham-operated rats, which reduced by treatment with nefopam, indicating that oligodendrocytes might respond to the peripheral nerve injury. However, less is known about the involvement of oligodendrocytes than astrocytes and microglial cells in neuroinflammation. A recent study demonstrated an important role of oligodendrocyte-derived interleukin (IL)-33 in neuropathic pain [27]. Oligodendrocytes in the spinal cord release IL-33 after chronic constriction injury, and IL-33 acts on IL-33 receptors (ST2) and the IL-1 receptor accessory protein (IL-1RAcP) expressed by endothelial cells, microglia, astrocytes, and neurons. Activation of the receptor complex triggers intracellular molecular signaling pathways such as phosphoinositide-3-kinase–protein kinase B (PI3K-PKB), mTOR, MAPKs (ERK, JNK, and p38), and nuclear factor κB (NF-κB), which are implicated in the production of IL-1β and TNF-α, and in development of neuropathic pain. TNF-α is implicated in the development of pro-inflammatory processes and neuropathic pain after nerve injury [28, 29]. We found that nefopam suppressed SNL-induced TNF-α production. Furthermore, lymphocytes infiltration into the spinal cord response to the SNL was found, indicating that immune responses might be involved in development of neuropathic pain. Indeed, in the spared nerve injury model of peripheral neuropathic pain, T-cell infiltration and activation in the dorsal horn of the spinal cord following peripheral nerve injury contribute to the evolution of neuropathic pain-like hypersensitivity [30]. Therefore, our present results suggest that nefopam may reduce proinflammatory cytokines by inhibiting oligodendrocyte activation and the immune response. However, further research is required to validate these findings.

Although, autophagic responses are known to help cells avoid death by offering an alternative cell-death pathway as a stress adaptation [31], the role of autophagy in the development of neuropathic pain is controversial. Shi et al. [32] reported that miRNAs regulate neuroinflammation and neuropathic pain through controlling autophagy. The level of miR-195 increased after SNL, which leads to change in autophagy and proinflammatory cytokine production in microglia, suggesting that miR-195/autophagy signaling involves in regulating neuroinflammation and neuropathic pain, and offering a new target for therapy of neuropathic pain. Recent report also showed blocking of basal autophagic turnover in the upregulated condition of autophagy after SNL may result in a degenerative pathway through the accumulation of dysfunctional macromolecules and organelles. Berliocchi et al. [2] suggested neuropathic pain after SNL is associated with disruption of autophagy by blocking autophagosome turnover. According to their results, the levels of LC3 and p62 has increased, and this accumulation of p62 represent impairment of normal autophagic process and blockade in the autophagic flux. On the contrary, the LC3-I levels has increased but p62 levels has decreased in our study, which represent increased activity of autophagy, not dysregulation. It is not clear why this discrepancy has developed, but the difference of the timing of harvesting spinal nerve (7 days from SNL vs. 14 days in our study), the species of animal used (mouse vs. SD rat in our study), or the methodology would be the reasons. Even though, autophagic activity inversely correlate with expression levels of p62, but it is not clear how p62 levels correlate with autophagy induction in vivo [33]. Moreover, autophagy may be upregulated as a response for stresses, but there is no clear evidence whether autophagic activity might be transcriptionally upregulated [33]. Because autophagic activity does not only depend on increased LC3-II, as well as on the coordination of regulatory proteins [2], confirmation through independent experiments such as morphologic evaluation is recommended to overcome these potential limitations [33]. In the present study, immunohistochemistry data show that increased autophagic activity by SNL was reduced by nefopam treatment, which was consistent with Western blot data. Suppression of autophagy may protect cells against neuroinflammation and relieve neuropathic pain development.

Based on our in vitro and in vivo studies, we speculate that the inhibitory effect of nefopam on neuropathic pain following SNL might be associated with regulation of the JNK-mediated autophagy signaling by nefopam. Although our present results show a relationship between autophagy and the effects of nefopam, however, it need to be improved the precise mechanisms of nefopam molecular regulation on autophagic processes and proinflammatory cytokines to ensure our hypothesis on the causal relationship between nefopam and autophagy.

## Conclusions

In conclusion, SNL increased autophagy and JNK activation, and also increased lymphocyte infiltration into the dorsal gray matter. Consequently, PWT significantly increased and neuropathic pain developed. However, nefopam injection markedly decreased autophagy and JNK activation. Nefopam increased PWT after SNL, and its mode of action appeared to be associated with the downregulation of phospho-JNK and autophagy.

## Abbreviations

H & E: Hematoxylin and eosin
IL: Interleukin
IL-1RAcP: IL-1 receptor accessory protein
JNK: C-Jun N-terminal kinase
LC3: Microtubule-associated protein 1 light chain
MAPK: Mitogen-activated protein kinase
mTor: Mammalian target of rapamycin
NMDARs: N-methyl-D-aspartate receptors
PI3K-PKB: Phosphoinositide-3-kinase–protein kinase B
PWT: Paw withdrawal threshold
SNL: Spinal nerve ligation
TNF-α: Tumor necrosis factor alpha
TRPV1: Transient receptor potential cation channel subfamily V member 1
